# Immunohistochemistry comparing endoscopic vein harvesting vs. open vein harvesting on saphenous vein endothelium

**DOI:** 10.1186/1749-8090-9-101

**Published:** 2014-06-17

**Authors:** Mohammad Hassan Nezafati, Pouya Nezafati, Sakineh Amoueian, Armin Attaranzadeh, Hamid Reza Rahimi

**Affiliations:** 1 Department Cardiac Surgery, Emam Reza Academic Hospital, Mashhad University of Medical Sciences, Mashhad, Iran; 2 Student Research Committee, Mashhad University of Medical Sciences, No 124., Niloufar 8th , Sadjad Blvd, Mashhad 91878, Iran; 3 Department of Pathology, Emam Reza Academic Hospital, Mashhad University of Medical Sciences, Mashhad, Iran; 4 Department of Molecular Pathology and Cytogenetics, Mashhad University of Medical Sciences, Mashhad, Iran; 5 Student Research Committee, Department of Modern Sciences & Technologies, School of Medicine, Mashhad University of Medical Sciences, Mashhad, Iran

**Keywords:** EVH, OVH, vWf, E-cadherin, Caveolin, eNOSs

## Abstract

**Objective:**

The present study attempts to compare the immunohistochemistry (IHC) of von Willebrand factor (vWf) , endothelial cadherin, Caveolin and endothelial Nitric Oxide Synthase (eNOS) in VasoView Endoscopic Vein Harvesting (EVH) versus traditional Open Vein Harvesting (OVH) techniques for Coronary Artery Bypass Graft (CABG) Surgery performed in *Javad al Aemeh* Hospital of Mashhad, Iran in 2013,.

**Methods and materials:**

Forty-seven patients were scheduled for CABG (30 EVH and 17 OVH) among whom patients with relatively same gender and similar age were selected. Three separate two cm vein samples were harvested from each patient’s saphenous vein. Each portion was collected from distal, middle and proximal zones of the saphenous vein. The tissues were deparaffinized, and antigen retrieval was done using EZ-retriever followed by an immunohistochemistry evaluation with vWf, e-cadherin, Caveolin and eNOS. In addition, demographic questioner as of Lipid profile, FBS, BMI, and cardiovascular risk factors were collected. Data analyses, including parametric and nonparametric tests were undertaken using the SPSS 16 software. A P value < 0.05 was regarded as statistically significant.

**Results:**

The mean age of the EVH and OVH groups were 63.76 ± 9.51 and 63.63 ± 8.31 years respectively with no significant difference between them (p = 0.989). In addition, there was no great difference between the EVH and OVH groups in lipid profile, DM, HTN, smoking history, CVA, and valvular dysfunction (P > 0.05). Qualitative report of vWf, e-cadherin, Caveolin and eNOS reveals no significant difference between the EVH and OVH (P > 0.05).

**Conclusion:**

This study indicates that VasoView EVH technique causes no endothelial damage in comparison with OVH. This study could be a molecular confirmation for the innocuous of EVH technique.

## Background

Many people around the world suffer from coronary artery disease (CAD) that causes an enormous morbidity and mortality. In order to rescue individuals with this disease there is the major and best operation/cardiac surgery known as coronary artery bypass grafting (CABG) [1]. This specified surgery is performed on individuals with each of followed diseases: 1) left main coronary artery disease, 2) 3-vessel disease, 3) 3-vessel disease in diabetics, 4) severely depressed heart function, and 5) heart conditions in addition to CAD e.g. replacement of valves or reconstruction of the heart muscle [1].

More than 300,000 CABG operations are performed in the North America annually [2]. Furthermore, it has been reported that over 10,000 patients require CABG every year in Iran [3]. However, complication rates, morbidity and mortality after CABG surgery are still expected to enhance despite advances in this field of the surgery to overcome CAD and rescue the patients [4], so surgeons should reduce the other risk factors of mortality and morbidity of CABG such as wound complications of saphenous vein.

Endoscopic greater saphenous vein harvesting (EVH) decreases the wound complications related with open techniques [5]. In order to decrease the considerable morbidity and wound complications associated with the extensive incisions made in traditional approach to vein harvest, minimally invasive techniques such as EVH is recommended [6]. Also using minimally invasive methods could reduce intra-operative complications [7,8]. EVH uses CO_2_ and an endoscope to harvest the saphenous vein by a single tiny incision [9].

In traditional methods of greater saphenous vein harvesting, large incisions must be taken; however, local pain in leg, dysmobility, wound infection, wound bleeding, prolonged hospital stay, and insufficient cosmetic results could happen [10,11].

A meta-analysis showed that EVH is safe and reduced rates of wound complications; leg wound infection, wound hematoma, and post operational pain, compared to traditional open techniques [6]. This research team in their previous study found EVH has fewer postoperative wound complications and less postoperative pain in comparison with OVH [8].

Allen KB et al. reported that “five-year follow-up of a prospective randomized clinical trial (RCT) display that use of EVH does not influence event-free survival” [5]. Some other scientists believe that EVH is independently associated with vein-graft failure [12]. Disruption of the endothelium by any instruments may cause the vein-graft failure so some heart surgeons still have doubts about EVH and they believe that it might cause damage to saphenous vein endothelium tissue [13]. Endothelial Nitric Oxide (eNOS), endothelial Caveolin (e-Caveolin), von Willebrand factor (vWf), and cadherin can evaluate endothelial function and damages [14]. Endothelial damage can be shown by displaced or decreased expression of eNOS and Caveolin [14]. vWF can demonstrate endothelial cell damage [15]. Serine/threonine protein kinase Akt can activate eNOS and endothelial dysfunction is due to the inadequate NO synthesis [16].

Although, vascular endothelial (VE)-cadherin is more specific to vascular endothelium but expression of this adhesion molecule might be an index for permeability and leukocyte extravasations. Therefore, we decided to evaluate e-cadherin [17].

The present study attempts to compare the immunohistochemistry of vWf, e-cadherin, Caveolin and eNOS in VasoView endoscopic saphenous vein versus traditional open vein harvesting techniques for CABG Surgery performed in *Javad al Aemeh* Hospital of Mashhad, Iran in 2013.

## Methods

From June 2013 to October 2013, 47 patients underwent CABG in Mashhad, Northeast of Iran. EVH performed on 30 patients and 17 had OVH. According to a preivouse research [13] and Daniel formula (Daniel 1999, Z value was 1.96, Expected proportion was 0.01, and Precision (d) was 0.0025) we used to determine the sample size of this study, so we needed 15.84 (≈16) cases in each group. Then we selected 17 controls in OVH and increased EVH group up to 30 cases.

This is a prospective cohort study, and demographic information (age, gender, educational level, marital status, income, and occupation), family history of CAD, smoking habits, FBS level and serum lipid profiles were almost similar in all patients.

All 47 patients had a same chance to be either in EVH or OVH group, they were numbered blindly, and then 17 numbers were selected from random number table for OVH and other 30 numbers were selected for EVH group.

As a primary choice for anastomosis, mammary arteries were used for the first vessel grafting in all 47 patients, and saphenous veins were used for the remaining number of anastomoses needed.

Three separate two cm vein samples were harvested from each of patient’s saphenous vein (distal, medial, proximal vein) for each EVH and OVH techniques, immediately after the harvesting.

### Endoscopic operative technique

Endoscopic dissection and excision of the saphenous vein has the advantage of requiring smaller skin incision, which heals better.

**Figure 1:**
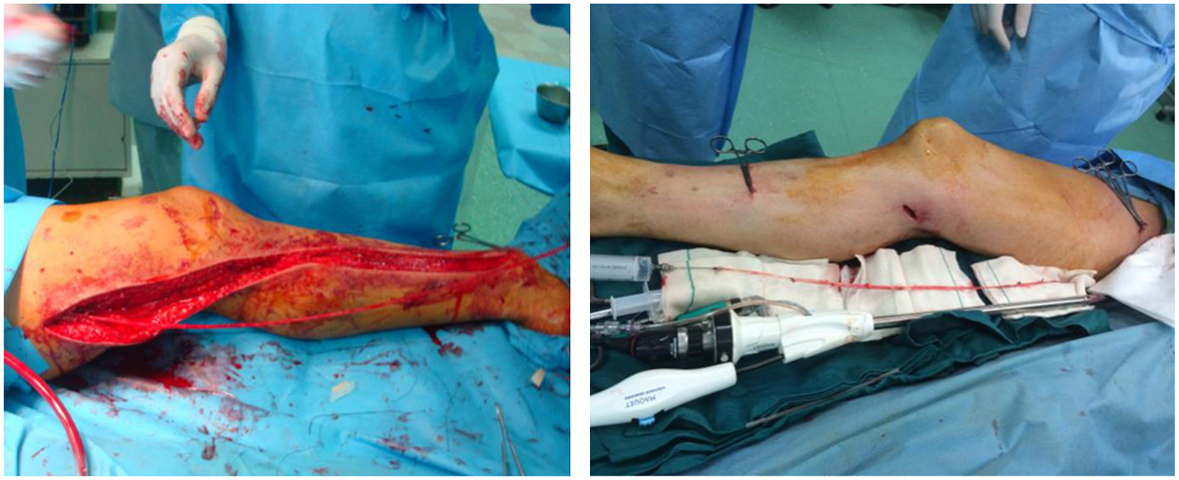
EVH and OVH procedure (EVH at right and OVH at left).

Our EVH method is based on the CO2 technique using a sealed and constant method of CO2 insufflations (VasoView HemoPro 2; Maquet^©^).A small incision is made 1.5-2.5 cm below/above the knee to build the entrance of the probe, which then continues its path toward the groin region. For dividing the branches, we used a bipolar cauterizer and by the use of a Scissor, a punctured incision was made to clamp, ligate and divide the vein. Ligation for small-size side branches was performed by clips whereas large-size branches and sites of basal disruption ligated with 7.0 monofilament prolene suture (this surgical team method), in addition to the application of clips for a greater blockage of the branches after harvesting the vein (Figure 1).

### Open operative technique

At first, the leg was abducted and rotated laterally by placing a roll under knee. After a long incision was made over the saphenous vein, side branches were ligated/clipped.The vein was then removed and prepared after the closure of the incision site in layers with absorbable suture and the leg wound was covered with cotton gauze dressing, in addition to applying an elastic ace to the entire leg (Figure 1).

### Immunohistochemical staining

**Figure 2:**
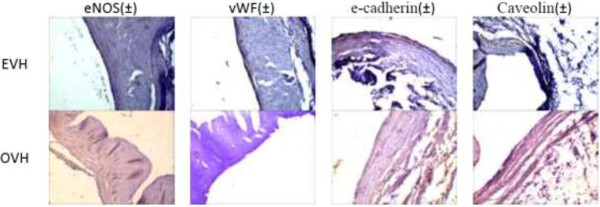
**eNOS, vWF, e-cadherin, and ccaveolin in EVH and OVH immunohistochemistry slides.** Qualitative assessing of slides were done by a same expert pathologist, as micrographs showed same staining intensity in intercellular and intracellular space.

Three micrometer thick tissue sections were placed on slides covered by Poly-L-Lysin, deparaffinized by xylol, and was dehydrated by graded alcohols for 10 minutes. Endogenous peroxidase activity was inhibited in H_2_O_2_-methanol solution. The de-paraffinized slides were rinsed with PBS and encountered with non-immune serum 10% for 10 minutes. Then cuts were incubated in a humidified chamber with an appropriate dilution of antibody (50 to 100 microliter) for 30–60 min at room temperature. Slides were washed in PBS for 5 min (Three replacements) and were incubated in a solution containing 3, 3 with 3 - amino -9 - ethyl. Counterstain was performed with Gill’s triple-strength hematoxylin. All these experimental procedures were performed under the instruction and protocols of Leica Biosystem (Novocastra) Corporation which is available in every immunohistochemistry kit. Immunohistochemistry staining was done for vWf, e-cadherin, Caveolin and eNOS. Negative staining reported by “-”, weakly positive staining reported by “, positive samples were reported by “ + ”, and finally strongly positive samples were reported by “++”. Single expert pathologist in IHC staining examined all samples (Figure 2).

### Statistical analysis

Statistical analysis used was the Statistical Package for Social Sciences version 16(SPSS Inc., Champaign, IL, USA). The Kolmogorov-Smirnov test was used to assess normality. Descriptive statistics (frequency, mean, and standard deviation) were determined for all variables. Values are reported as mean ± SD for normally distributed variables (or Median and Interquartile range (IQR) for not normal distribute variables). Baseline demographics and clinical characteristics were compared between groups using t-student test, chi-square test, Friedman test, and/or Fisher exact test as appropriate. A p value < 0.05 was considered as significant.

## Results

Mean age has no significant difference between EVH and OVH 63.76 ± 9.51 and 63.63 ± 8.31 respectively (P = 0.961). In addition, there is no noticeable difference between groups in gender, smoking, hypertension, hyperlipidemia, CVA history, BMI, and family history of CAD (P > 0.05) (Table 1).

**Table 1:** Characteristics data from all subjects in each group

**Variable**	**EVH (n = 30)**	**OVH (n = 17)**	**P value**
Age (year)	63.76 ± 9.51	63.63 ± 8.31	0.961
Sex			
Male	23(76.7)	11(64.7)	0.378
Female	7(23.3)	6(35.3)	
Smoking			
Yes	9(32.1)	7(41.2)	0.539
No	19(67.9)	10(58.8)	
Diabetes mellitus			
Yes	14(51.9)	2(11.8)	0.007
No	13(48.1)	15(88.2)	
Hypertension history			
Positive	16(59.3)	11(64.7)	0.718
Negative	11(40.7)	6(35.3)	
Hyperlipidemia history			
Positive	15(53.6)	8(47.1)	0.672
Negative	13(46.4)	9(52.9)	
CVA (stroke) history			
Positive	0(0.0)	0(0.0)	-
Negative	28(100)	17(100)	
Family history of CAD			
Positive	8(26.6)	4(23.5)	0.931
Negative	22(74.4)	13(76.5)	
Valvular disease comorbidity			
Positive	2(7.1)	2(11.8)	0.597
Negative	26(92.9)	15(88.2)	
CABG reason			
2VD	1(3.6)	2(11.8)	0.285
3VD	27(96.4)	15(88.2)	
BMI	27.50 ± 4.93	26.00 ± 3.86	0.291
BUN	35.78 ± 20.41	29.85 ± 14.67	0.346
Creatinine	0.99 ± 0.27	1.09 ± 0.25	0.240
FBS (mg/dl)	136.0 ± 55.83	111.81 ± 36.1	0.128
TC (mg/dl)	158.11 ± 45.68	207 ± 122.73	0.063
LDL-C (mg/dl)	69.77 ± 36.50	94.07 ± 40.21	0.058
HDL-C (mg/dl)	51.25 ± 13.56	50.85 ± 6.13	0.917
TG (mg/dl) (median(IQR))	165(72)	175(85)	0.294

Date are shown as Number (percent), or mean ± SD, or (median (IQR)).

CVA: cerebrovascular accident, CABG: Coronary artery bypasses surgery, BMI: Body Mass Index, BUN: blood urea nitrogen, FBS: Fasting blood sugar, TC: total cholesterol, LDL-C: Low density lipoprotein cholesterol, HDL-C: High-density lipoprotein, TG: triacylglyceride, CAD: Coronary artery disease, 2VD: two-vessel coronary artery disease, 3VD: three-vessel coronary artery disease.

Most of the patients were referred to our center due to three-vessel coronary disease (Table 1).

BUN, Cr, FBS, total cholesterol, LDL-C, HDL-C, and triglyceride had no significant difference between EVH and OVH group (P > 0.05) (Table 1).

Multivariate regression analysis demonstrated no influence of basic characteristics variable on IHC outcome.

According to Table 2, eNOS, E-cadherin, Caveolin, and vWF immunohistochemistry staining in distal, medial and proximal zones of vein samples in both groups had no significant statistical difference (P > 0.05) (Table 2) (Figure 2).

**Table 2:** Immunohistochemistry results from all subjects in each group

**Variable**			**EVH**	**OVH**	**P value**	
			**(n = 30)**	**(n = 17)**		
	Distal	±	23(76.7)	12(70.6)	0.646	P* = 0.513
		+	7(23.3)	5(29.4)		P** = 0.882
		±	21(77.8)	8(53.3)		
eNOS	Medial	+	5(18.5)	7(46.7)	0.131	
		++	1(3.7)	0(0.0)		
	Proximal	±	22(73.3)	12(70.6)	0.840	
		+	8(26.7)	5(29.4)		
Caveolin	Distal	±	22(73.3)	12(70.6)	0.840	P* = 0.779
		+	8(26.7)	5(29.4)		P** = 0.180
		±	16(59.3)	12(80.0)		
	Medial	+	10(37.0)	3(20.0)	0.353	
		++	1(3.7)	0(0.0)		
		±	21(70.0)	14(82.4)		
	Proximal	+	8(26.7)	3(17.6)	0.558	
		++	1(3.3)	0(0.0)		
E- Cadherin	Distal	±	21(70.0)	14(82.4)	0.351	P* = 0.097
		+	9(30.0)	3(17.6)		P** = 0.509
		-	1(3.7)	0(0.0)		
	Medial	±	14(51.9)	10(71.4)	0.424	
		+	12(44.4)	4(28.6)		
		±	20(66.7)	13(76.5)		
	Proximal	+	9(30.0)	4(23.5)	0.644	
		++	1(3.3)	0(0.0)		
		±	2(6.7)	1(5.9)		P* = 0.368
	Distal	+	27(90.0)	16(94.1)	0.742	P** = 0.417
		++	1(3.3)	0(0.0)		
		±	3(10.7)	1(7.1)		
vWF	Medial	+	21(75.0)	12(85.7)	0.718	
		++	4(14.3)	1(7.1)		
		±	3(10.0)	3(17.6)		
	Proximal	+	23(76.7)	13(76.5)	0.588	
		++	4(13.3)	1(5.9)		

Date are shown as Number (percent).

NOS: nitric oxide synthase, vWF: Von Willebrand factor.

P*: P value of Friedman test for comparing distal, medial, and proximal sample within group of OVH.

P**: P value of Friedman test for comparing distal, medial, and proximal sample within group of EVH.

Hospital follow-ups were performed including echocardiography, CKB-MB and Troponin cardiac enzymes. A rise of double for CKB- MB and Troponin detected in one patient who underwent OVH. This rise was managed to the normal range in the period of hospital stay and no wall motion abnormality or EF changing appeared in echocardiography.

Finally, we have compared three samples taken from each person in both groups, which were analyzed by Friedman test, and we found no significant difference between IHC results in same patient in different parts of vein (P > 0.05) (Table 2). In simple H&E histological slides, there was no structural and morphological difference between groups.

## Discussion and conclusion

Chronic wounds or post operation complicated wounds are associated with increased morbidity and mortality and pose a serious economic burden on the health-care system. It has been estimated that nearly $25 billion is spent annually in the United States to treat ulcers [18].

These chronic and non-healing wounds are the ones in which the healing progress is less than (20-40% reduction in the area) after 2–4 weeks of appropriate approach and treatment [19].

Traditional greater saphenous vein harvesting may increase the post operation wound complications [20]. These wounds and their complications can influence the patient’s physical activity and may make limitations for patients, so it is very harmful in post CABG rehabilitation therapy [21]. Surgeons’ fatigue before they reach to the main part of the operation procedure is crucial; however, a great amount of time is gained for skin closure in OVH technique [22] whereas, EVH is performed at a satisfactory shorter time [23]. Considering all of these points, minimal invasive vein harvesting is noticeable.

Some surgeons and scientists believe that EVH may destroy greater saphenous vein tissue and it could be harmful for bypass grafting [12]. In this study, we aimed to compare the IHC tissue staining result between EVH and OVH greater saphenous vein.

According to our results, there were no significant differences between same site samples in EVH and OVH between patients and no noticeable difference in distal, medial and proximal sites within a patient sample was detected in eNOS, E-cadherin, Caveolin, and vWF.

Bader E. Hussain et al. in 2011 reported that EVH preserve the structural and functional viability of greater saphenous vein [13]. They found that western blots, immunofluorescent, multiphoton imaging had no difference for caveolin 1, eNOS, Cadherin, and vWf between EVH and OVH [13].

There were no changes in caveolin and eNOS in the vein endothelial in EVH technique in this study. These results are very important for evidence-based decision and in molecular view, there are no structural and cellular viability harm effect found in EVH compared with OVH.

Iamar M. Al Saggaf et al. found statistically insignificant difference between EVH and OVH by scanning electron microscopy (SEM) assay [24]. In other histological studies, there were minimal and negligible changes between EVH and OVH in light and electronically microcopy [25-28].

### Limitations

Matching patients with similar criteria as of DM was our limitation.

### Ethical issue

The study was conducted in accordance with the principles of Declaration of Helsinki 1996 version and Good Clinical Practice standards. All subjects signed informed-consent forms.

## Abbreviations

IHC: Immunohistochemistry; vWf: Von Willebrand factor; eNOS: Nitric oxide synthase; EVH: Endoscopic vein harvesting; OVH: Open vein harvesting; CABG: Coronary artery bypass graft; CAD: Coronary artery disease; RCT: Randomized clinical trial; e-Caveolin: Endothelial caveolin; IQR: Interquartile range; CVA: Cerebrovascular accident; BMI: Body mass index; BUN: Blood urea nitrogen; FBS: Fasting blood sugar; TC: Total cholesterol; LDL-C: Low density lipoprotein cholesterol; HDL-C: High-density lipoprotein; TG: Triacylglyceride; 2VD: Two-vessel coronary artery disease; 3VD: Three-vessel coronary artery disease; HTN: Hypertension; DM: Diabetes mellitus; SEM: Scanning electron microscopy.

## Competing interest

The authors declare that they have no competing interests.

## Authors’ contributions

MHN: Head of research team & Research designer. PN: Data analysis & Data gathering & Patient follow up. SA: Pathology section. AA: Pathology section. HRR: Pathology section & Data analysis. All authors read and approved the final manuscript.
